# The postcard intervention against depression among community-dwelling older adults: study protocol for a randomized controlled trial

**DOI:** 10.1186/1745-6215-14-202

**Published:** 2013-07-09

**Authors:** Hissei Imai, Toshiaki A Furukawa, Kiyohito Okumiya, Taizo Wada, Eriko Fukutomi, Ryota Sakamoto, Michiko Fujisawa, Yasuko Ishimoto, Yumi Kimura, Wen-ling Chen, Mire Tanaka, Kozo Matsubayashi

**Affiliations:** 1 Field Medicine, Graduate School of Medicine/School of Public Health, Kyoto University, Yoshida Konoe-cho, Sakyo-ku, Kyoto, 606-8501, Japan; 2 Departments of Health Promotion and Human Behavior and of Clinical Epidemiology, Kyoto University Graduate School of Medicine/School of Public Health, Yoshida Konoe-cho, Sakyo-ku, Kyoto, 606-8501, Japan; 3 Institute for Humanity and Nature, 457 Kamigamo Motoyama, Kita-ku, Kyoto, 603-8047, Japan; 4 The Center for Southeast Asian Studies, Kyoto University, 46 Yoshida Simoadachi-cho, Sakyo-ku, Kyoto, 606-8501, Japan; 5 Hakubi Center, Kyoto University, Yoshida Ushinomiya-cho, Sakyo-ku, Kyoto, 606-8302, Japan

**Keywords:** Depression, Non-clinical intervention, Prevention, Community, Older adults

## Abstract

**Background:**

Depression in older adults deteriorates quality of life and increases morbidity, mortality, and medical expenses. Medicine and social policy should work together to decrease this burden. Existing prevention studies are often based on time-consuming psychotherapies, which therefore are not feasible for a wide application at the community level. Postcard interventions have been shown to be effective for patients after hospitalization for major depression, drug overdose, or self-harm. This paper describes the protocol of a pragmatic, randomized controlled trial designed to examine the efficacy of a postcard intervention for depression among community-dwelling individuals aged 65 years or older.

**Methods/Design:**

This is a pragmatic, non-blinded, parallel comparison, randomized controlled trial using Zelen’s design in a community setting. Participants will include community-dwelling older adults (aged 65 years or older) with limited social support (indicated by eating meals alone) and with symptoms of depression (scoring 4 or higher on the 15-item Geriatric Depression Scale (GDS)). The intervention will consist of sending postcards with handwritten messages and seasonal reports from a historical city to participants once a month for eight consecutive months. Self-addressed, stamped envelopes will be enclosed to facilitate non-obligatory replies. Primary outcomes will be changes in the GDS scores that are administered to all elderly inhabitants of the community every year as part of annual health checks. Secondary outcomes include quality of life as measured by a visual analogue scale, and self-rated basic and advanced activities of daily living. We will also examine the subjective sense of effectiveness of the intervention, recollection of the number of intervention mailings received, and the number of mailed replies as the index of the acceptability of the postcard intervention. The time × group interaction for two consecutive years will be analyzed using a generalized linear mixed model. To detect an effect size of 0.5 at alpha error of 0.05 and statistical power of 0.80, 63 participants per group are required. Based on an estimated consent and dropout rate of 70%, a total of 180 subjects will be recruited.

**Trial registration:**

UMIN000010529

## Background

Depression is frequent and chronic in older adults. According to research on community-dwelling older adults, the proportion of individuals reporting depressive symptoms is 2.8% to 35% [1]. The natural course of later-life depressive disorders is poor: a 6-year follow-up study showed that 76% of patients followed an unfavorable but fluctuating course or a severe chronic course of depression, and only 23% of patients experienced full remission [2].

Depression in older adults deteriorates the sufferers’ quality of life (QOL) more than many other chronic diseases [3]. It gives a negative impact on patients’ QOL in various ways, including wellbeing, perceived physical functioning, bodily pain, and general health perceptions [4]. The mortality rate of people with depression was found to be 1.8 times larger than that of non-depressed subjects due to suicide, unhealthy habits, and medical illnesses [5].

Depression is also costly. Depressed older adults use more outpatient resources than those without depression, including frequent appointments and increased laboratory and radiographic tests. They also have more non-specific medical complaints, and this is associated with increased total ambulatory care costs [6]. A study in the United States found that the additional medical cost per one depressed older adult was USD 686 for 1 year and USD 5,271 for 4 years [7].

As the world population continues to age, there is an urgent need therefore for medicine and social policy to find ways to reduce and prevent depression in older adults in the community.

However, to the best of the authors’ knowledge, no simple, effective interventions currently exist for the prevention of depression in the elderly population [8]. The existing prevention studies have limitations in study design or rely on time-consuming psychotherapy, which is unrealistic for a community prevention program. They need weekly sessions with a duration of 45 to 120 minutes for 6 to 10 weeks [9-12], and trained workers or specialists [9,11-14]. The subjects of most of the studies were not general people in community but those with specific disease or physical symptoms such as diabetes [10], macular degeneration [11], hip fracture [15], chronic pain [12], and most of the studies recruited subjects in clinical settings [10,11,13,15]. Some studies lacks sample size calculation [9,10,12] and were quasi-randomized controlled trials [10,14].

A postcard intervention was first carried out in the United States in 1976 for suicide prevention among discharged major depression patients. Researchers sent 24 letters over 5 years and reported that this significantly decreased suicide rates for the first 2 years and tended to lower suicide rates up to 13 years in total [16,17]. Three more postcard intervention trials were conducted in Israel and Australia in 2005, 2010, and 2011, that focused on the prevention of drug overdose or self-harm. The results showed significant decrease in the number of drug overdose episodes, and the rates of suicide ideation and suicide attempts [18-21]. The prevention of depression in patients with a recent stroke by postcard is also planned [22].

The advantage of the postcard intervention is its low personal and financial cost: it only requires paper, pencil, and postage. Therapists are not required to visit the participants and vice versa. If the postcards do not contain medical and related information, a wide range of people such as elementary school students can take part in the intervention program.

This paper describes the study protocol for a pragmatic, randomized controlled trial designed to examine the effectiveness of the postcard intervention for improvement of depression in community-dwelling individuals aged 65 years or older. This study will focus in particular on those who have increased depressive symptoms and insufficient social support at baseline, because it is expected that the intervention is more effective among such individuals.

### Objectives

For community-dwelling older adults (aged 65+ years) reporting symptoms of depression and limited social support, this study aims to: (1) examine the effectiveness of a postcard intervention for the improvement of depressive symptoms; (2) evaluate the effectiveness of a postcard intervention in global geriatric health indicators such as quality of life (QOL) and the activities of daily living (ADL); and (3) assess the acceptability of the postcard intervention.

## Methods and design

### Ethical approval

The Institutional Review Board (IRB) of the Graduate School of Medicine, Kyoto University has reviewed and approved the study protocol and informed consent documents (E1658, 12 February 2013).

### Study setting

The study will be conducted in the community of a rural town, located in the center of Shikoku, one of the four main islands in Japan. Its main industries are agriculture and forestry. It has a population of 4,407, of whom 1,711 (38.8%) are aged 65 years or older.

Our study team has been conducting a longitudinal observational study in this community since 2004, in which we administer comprehensive geriatric assessments and report results and make referral to physicians as necessary. This observational study has been approved by the IRB of the Graduate School of Medicine, Kyoto University (E-18), and written informed consent has been obtained from all the participants.

### Study design

#### Design overview

**Figure 1:**
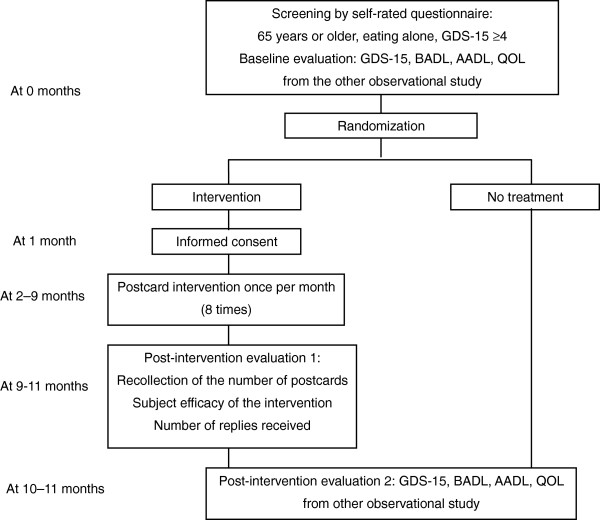
**Participant flowchart.** GDS-15: 15-item Geriatric Depression Scale; AADL: activities of daily living; BADL: basic activities of daily living; QOL: quality of life.

We will conduct a pragmatic, non-blinded, parallel comparison, randomized controlled trial using Zelen’s design in this community. Figure 1 depicts the participants’ flow. Participants will be selected based on the questionnaire surveys including a self-rated depression scale. Participants will then be randomized to the intervention or no-intervention groups at a 1:1 ratio using computer-generated random numbers. Randomization will be stratified by gender and self-rated depression scale score. To conceal group assignments, random number generation and group allocation will be conducted at the same time by an independent epidemiologist not involved in the participant recruitment or intervention or assessments. Informed consent forms will be mailed to those assigned to the intervention group. Postcards will be sent to the consenting participants once a month for eight consecutive months. They will be enclosed with a self-addressed stamped envelope to facilitate non-obligatory replies by mail. As this is a pragmatic study, any treatment outside the trial will be permitted. Self-reported outcomes will be measured at baseline and post intervention; the questionnaire about participants’ impression of effectiveness of the intervention and their recollection of the number of postcards received will be measured after treatment.

#### Zelen’s design

The study uses a randomized controlled trial with the single consent version (Zelen’s design) [23,24]. This is a variation of the standard randomized controlled design in which participants are randomized to intervention or control before consent is sought. Consent is obtained from the intervention group only after the randomization. The most important advantage of this method is that participants know the intervention they will receive at the time of consent. In a conventional randomization, participants who agree to join the study may retract their consent or continue participation with reluctance after finding out their assigned intervention, whereas the Zelen’s method requires a decision only on the allocated intervention.

The main ethical concern is that consent is obtained only from the intervention group. To overcome this point, the revised Zelen method has been proposed [25]. This method is a combination of an observational study and a randomized controlled trial. Eligible participants first consent to an observational study, and then they are randomly assigned to intervention and control groups; those in the intervention group are asked to consent to participate in the study. Those in the control arm are not informed of this, but will be followed in the observational study if they agreed. Our protocol will follow this method.

### Participants

#### Inclusion criteria

Participants will meet the following criteria: (1) being 65 years of age or older; (2) exhibiting symptoms of depression with a score of ≥4 on the self-rated 15-item Geriatric Depression Scale (GDS-15); and (3) reporting that they eat meals alone in the questionnaire.

The study will include individuals with sub-threshold depression. Indicated prevention aimed at sub-threshold depression is said to be most efficient in terms of costs and benefit [26]. As there are no agreed-upon definitions of sub-threshold depression on GDS-15, the study will include those with scores ≥4 points on the GDS-15, which is 1 point below the established cutoff to detect major depression [27].

Considering the nature of the intervention, the study will target those who are at risk of social isolation. We hypothesize that eating alone, rather than living alone, better represents the risk of isolation. In fact, eating alone was more strongly associated with depression than living alone among community-dwelling older adults [28].

#### Exclusion criteria

Participants will be excluded if they cannot understand and sign the informed consent form. Those who currently reside in a hospital or institution will be excluded.

### Sample size

To detect an effect size of 0.5 with *P* = 0.05 at 80% power, 63 participants are required per group. Assuming a non-consent and dropout rate of 30%, a total of 180 subjects are needed. Based on the results of our previous observational study performed in the same town in 2012, this sample size is believed to be feasible.

### Intervention

Letters written on A4 paper with some colorful illustrations will be sent in a sealed envelope once a month for 8 months. The letter will be composed of two parts: the first part will be a handwritten reply to messages returned from the participants if there are replies or comments, which aims to increase social connectedness and to enhance their self-respect; the second part will be seasonal greetings or news of the month from Kyoto, Japan, where the study authors are located, printed by computer. Kyoto is one of the most famous cultural centers in Japan and hosts various historical events that we believe will be of interest to participants living far from Kyoto.

Although a self-addressed stamped reply card will be enclosed with the letter, replying is not mandatory; this will be indicated on the reply card.

### Outcomes

#### Primary outcomes

Primary outcomes will be the change in GDS-15 score as the measure of effectiveness.

#### Secondary outcomes

Secondary outcomes will be self-rated QOL as evaluated by visual analogue scales, self-rated basic ADL, and self-rated advanced ADL.

#### Other outcomes

The subjective sense of effectiveness of the intervention, recollection of the number of intervention mailings received, and the number of mailed replies will be evaluated to measure acceptability of the postcard intervention.

### Outcome measures

#### GDS-15

The GDS-15 is a validated depression scale comprised of 15 items. This scale was developed to exclude the effects of non-specific somatic symptoms such as anorexia and insomnia, which are frequently observed among elderly populations [29,30]. Each item can have two answers: yes or no. The highest possible score is 15, indicating the most severe depressive state. Using a cutoff point of 5, the GDS-15 has a sensitivity of 92% and a specificity of 81% to detect major depression as ascertained by a structured clinical interview [27].

#### QOL

Subjective QOL will be assessed using a 100-mm visual analogue scale (lowest QOL on the left end of the scale, and highest on the right) for the following five items: subjective sense of health; relationship with family; relationship with friends; financial state; and subjective happiness [31,32].

#### Basic ADL (BADL)

Each participant will rate his or her independence with respect to seven items corresponding to basic activities of daily living (BADL). Specifically, these items are as follows: walking, ascending and descending stairs, feeding, dressing, going to the toilet, bathing, and grooming. Each BADL item is evaluated based on four levels of competence: 3, completely independent; 2, requiring some assistance; 1, requiring much assistance; 0, completely dependent. The scores for the seven BADL items will be summed for a total score of 0 to 21 [33,34].

#### Advanced ADL (AADL)

For higher-level functional capacity, the Tokyo Metropolitan Institute of Gerontology Index of Competence rating scale will be used to measure competence [35,36]. This scale consists of 13 items encompassing three sublevels of competence: (1) instrumental ADL (five items: the ability to use public transport, buy daily necessities, prepare a meal, pay bills, and handle banking matters); (2) intellectual activities (four items: the ability to complete forms, read newspapers, read books or magazines, and show interest in television programs or news articles on health-related matters); and (3) social roles (four items: the ability to visit friends, give advice to relatives and friends in confidence, visit someone at the hospital, and initiate conversation with younger people). Because each item is rated as ‘yes’ or ‘no’, instrumental ADL has a score range of 0 to 5, intellectual ADL 0 to 4, and social role ADL 0 to 4.

#### Sociodemographic and other information

Data about age, sex, eating alone, and living alone will be obtained through a self-reported questionnaire. Participants’ subjective sense of the effectiveness of the intervention will be evaluated on a four-point scale ranging from 0 (not effective) to 4 (very effective).

### Management of adverse events

We expect that no adverse events will result from the postcards. However, if an emergent situation such as a high risk of suicide is suspected based on the reply card, a certified psychiatrist will evaluate the participant and refer him/her to the hospital if needed.

### Statistical analysis

The time × group interaction for baseline and post intervention will be analyzed using a generalized linear mixed model, which enable us to analyze data even when they have missing values. Sensitivity analysis will be conducted by way of ANCOVA with data imputed by a multiple imputation method and with completer’s data, using baseline data alone or with ADL score as a covariate and post-intervention data as dependent variables. The homogeneity of variance will be analyzed with Leven’s test. Statistical analysis will be performed using SPSS ver. 20.0 (IBM Inc., Armonk, NY, USA).

## Discussion

The study protocol describes the design of a pragmatic randomized controlled trial to verify the efficacy of postcard intervention to prevent and improve depression among community-dwelling older adults in Japan. This is the first application of the postcard intervention for depression of community-dwelling older adults. The advantage of the postcard intervention is its low human and financial cost, which cannot be matched by other existing approaches such as psychotherapy. This intervention can be carried out by anyone who can write a letter. Its application will be broad.

There are three advantages to the study. First, the study is set in the community, whereas previous postcard interventions were conducted in clinical settings [16-19,21]. Considering the potential of postcard interventions, however, application to a broader field is desirable; the application in community settings targeting local residents is but one of them. Second, the study focuses on people who eat alone, not those who live alone. The supposed effect of postcard intervention is to make a connection with people, thereby reducing feelings of isolation. Even if a person is surrounded by many other people, he or she will be lonely unless people pay attention to him or her. In this sense, eating alone rather than living alone can reflect true isolation [28]. Third, the study will evaluate ADLs. Previous prevention studies did not consider their effect on ADLs. However, as the participants in this study are older adults whose ADLs have bidirectional interactions with depressive mood, the influence of ADLs should be considered and the effects of the intervention on AADL in particular should be evaluated.

If the efficacy of the postcard intervention for depression in community-dwelling older adults is verified, it will be a milestone in community intervention.

## Trial status

Participant recruitment will begin in June 2013.

## Abbreviations

GDS-15: 15-Item geriatric depression scale; AADL: Advanced activities of daily living; ADL: Activities of daily living; BADL: Basic activities of daily living; IRB: Institutional review board; QOL: Quality of life; VAS: Visual analogue scale.

## Competing interests

TAF has received honoraria for speaking at CME meetings sponsored by Asahi Kasei, Eli Lilly, GlaxoSmithKline, Mochida, MSD, Otsuka, Pfizer, Shionogi, and Tanabe-Mitsubishi. He is a diplomate of the Academy of Cognitive Therapy. He has received royalties from Igaku-Shoin, Seiwa-Shoten, and Nihon Bunka Kagaku-sha. He is on the advisory board for Sekisui Chemicals and Takeda Science Foundation. The Japanese Ministry of Education, Science, and Technology, the Japanese Ministry of Health, Labor and Welfare, and the Japan Foundation for Neuroscience and Mental Health have funded his research projects. All the other authors report no competing interests.

## Authors’ contributions

HI and KM made substantial contribution to the conception and design of the study, were involved in drafting the manuscript, and will be responsible for the administration and direction of the study as well as the analysis and interpretation of data. TAF made a substantial contribution to the conception of the study, was involved in drafting the manuscript, and will be responsible for the analysis and interpretation of data. TF, KO, and EF assisted with the negotiation with the local government, and will be responsible for the preparation of the study materials as well as the analysis and interpretation of data. YI, YK, WC, and MM will be responsible for data collection. RS contributed to the conceptualization of the study design and will be responsible for data analysis and interpretation. All authors read and approved the final manuscript.
